# Polyglutamine binding protein 1 regulates neurite outgrowth through recruiting N-WASP

**DOI:** 10.1016/j.jbc.2024.107537

**Published:** 2024-07-04

**Authors:** Xuejiao Huang, Shanshan Cheng, Junhai Han

**Affiliations:** 1 School of Life Science and Technology, The Key Laboratory of Developmental Genes and Human Disease, Southeast University, Nanjing, China; 2 Co-innovation Center of Neuroregeneration, Nantong University, Nantong, JS, China

**Keywords:** polyglutamine binding protein 1, neurite outgrowth, N-WASP, growth cone, protein interaction, peptide interference

## Abstract

Neurite outgrowth is a critical step in neural development, leading to the generation of neurite branches that allow individual neurons to make contacts with multiple neurons within the target region. Polyglutamine-binding protein 1 (PQBP1) is a highly conserved protein with a key role in neural development. Our recent mass spectrometric analysis showed that PQBP1 associates with neural Wiskott-Aldrich syndrome protein (N-WASP), an important actin polymerization-promoting factor involved in neurite outgrowth. Here, we report that the WW domain of PQBP1 directly interacts with the proline-rich domain of N-WASP. The disruption of this interaction leads to impaired neurite outgrowth and growth cone size. Furthermore, we demonstrate that PQBP1/N-WASP interaction is critical for the recruitment of N-WASP to the growth cone, but does not affect N-WASP protein levels or N-WASP-induced actin polymerization. Our results indicated that PQBP1 regulates neurite outgrowth by recruiting N-WASP to the growth cone, thus representing an alternative molecular mechanism *via* which PQBP1-mediates neurite outgrowth.

Mutations in the polyglutamine-binding protein 1 (*PQBP1*) gene have been associated with Renpenning syndrome, an X-linked disorder characterized by intellectual disability, microcephaly, short stature, and specific facial dysmorphism (1, 2, 3, 4, 5). Studies in animal models and cultured cells have revealed that PQBP1 plays an essential role in neural development (6, 7, 8, 9, 10, 11). The conditional knockout (cKO) of the *Pqbp1* gene in mouse neural stem progenitor cells leads to the prolongation of the cell cycle and results in a microcephalic phenotype (6), while the depletion of *Pqbp1* gene in striatal progenitors results in reduced proliferation and increased differentiation of striatal progenitors (9). Moreover, the knockdown of PQBP1 in neurons was shown to result in substantially reduced dendrite branching and length (10). The application of full-length PQBP1 but not a mutated form of the protein reduced the defects in neurite outgrowth (10). The *PQBP1* gene encodes a widely expressed nucleocytoplasmic shuttling protein with multiple functions in both the cytoplasm and the nucleus (6, 7, 9, 10, 12). For instance, one study identified a link between PQBP1-mediated mRNA alternative splicing and neurite outgrowth (10). In our recent mass spectrometric analysis, we found that PQBP1 associates with neural Wiskott-Aldrich syndrome protein (N-WASP), a key actin polymerization-promoting factor involved in neurite outgrowth. However, whether PQBP1/N-WASP interaction is necessary for neurite outgrowth is unknown.

Neurite outgrowth is an essential part of neural differentiation and maturation and begins with the protrusion of the neuronal membrane, a process that largely depends on actin dynamics and cytoskeleton rearrangements (13, 14, 15). N-WASP is a critical regulator of actin dynamics, triggering Arp2/3-mediated actin nucleation. This promotes actin polymerization, thereby initiating the outgrowth of new daughter filaments and facilitating branching (16, 17, 18, 19, 20, 21). N-WASP possesses a WASP-homology domain 1 (WH1 domain) at N-terminus, followed by a basic region (BR), a Cdc42/Rac GTPase binding domain (GBD), a proline-rich domain (PRD), and a verprolin-cofilin-acidic (VCA) domain at the C-terminus (22, 23, 24). Studies have shown that N-WASP is activated by Cdc42 and phosphatidylinositol 4,5-bisphosphate, leading to the exposure of the VCA domain of N-WASP. This is followed by the recruitment of the Arp2/3 complex and the initiation of actin polymerization (21, 24, 25, 26). Although the regulation of N-WASP activation has been extensively studied, how N-WASP is recruited to the growth cone and subsequently induces neurite branching remains largely unclear.

Here, we show that PQBP1 co-localizes with N-WASP and directly interacts with it through its PRD. The disruption of this interaction does not affect the N-WASP protein level nor N-WASP-induced actin polymerization, but instead impairs the recruitment of N-WASP to the growth cone, resulting in defective neurite outgrowth and growth cone smaller. Our results reveal a novel cytoplasmic role for PQBP1, in which PQBP1 regulates neurite outgrowth by directly interacting with and thereby recruiting N-WASP to the growth cone. Our study provides an alternative molecular mechanism by which PQBP1 mediates neurite outgrowth.

## Results

### PQBP1 interacts with the cytoskeletal regulator N-WASP through its WW domain

**Figure 1:**
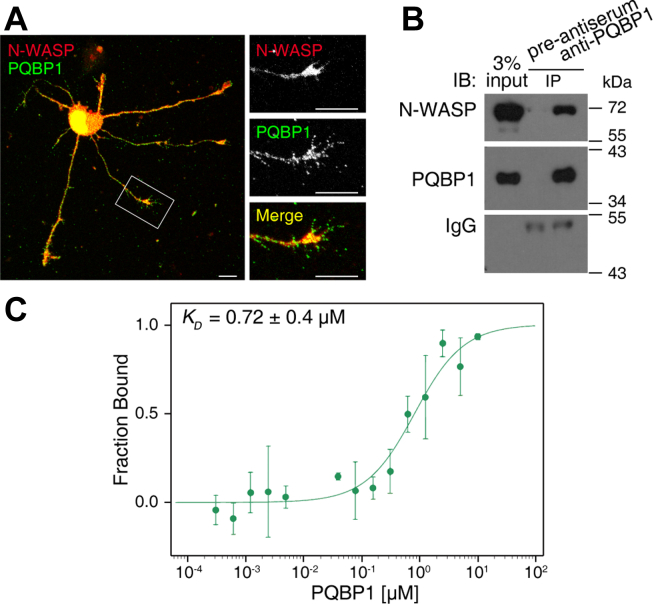
**PQBP1 directly binds to N-WASP.***A*, immunostaining images showing the co-localization of N-WASP with PQBP1 in cultured hippocampal neurons from wild-type mice at 2 days *in vitro* (2 DIV). Scale bar, 10 μm. *B*, co-immunoprecipitation of PQBP1 and N-WASP in neurons. Cytoplasmic extracts from 8–10-day-old mouse brains were immunoprecipitated with anti-PQBP1 antibodies or pre-antiserum. *C*, measurement of the PQBP1/N-WASP binding affinity. Data are presented as means ± SD of three independent experiments.

In our recent study, we identified several PQBP1-associated proteins using mass spectrometric analysis. Among these proteins, N-WASP, also known as actin nucleation-promoting factor WASL, was one of the top hits. To validate the co-localization between PQBP1 and N-WASP, we performed immunostaining in cultured mouse hippocampal neurons. The results revealed that PQBP1 largely co-localized with N-WASP in the nucleus as well as in neurite branches and termini (Fig. 1*A*). To confirm the physical association between PQBP1 and N-WASP in the cytoplasm *in vivo*, we carried out co-immunoprecipitation assays on cytoplasmic fractions of neurons isolated from the brains of mice. The results demonstrated that there was indeed an association between PQBP1 and N-WASP in the cytoplasm of cells *in vivo* (Fig. 1*B*). We further measured the binding affinity between PQBP1 and N-WASP using microscale thermophoresis analysis, and obtained a *K*_D_ value of 0.72 ± 0.4 μM (Fig. 1*C*).

**Figure 2:**
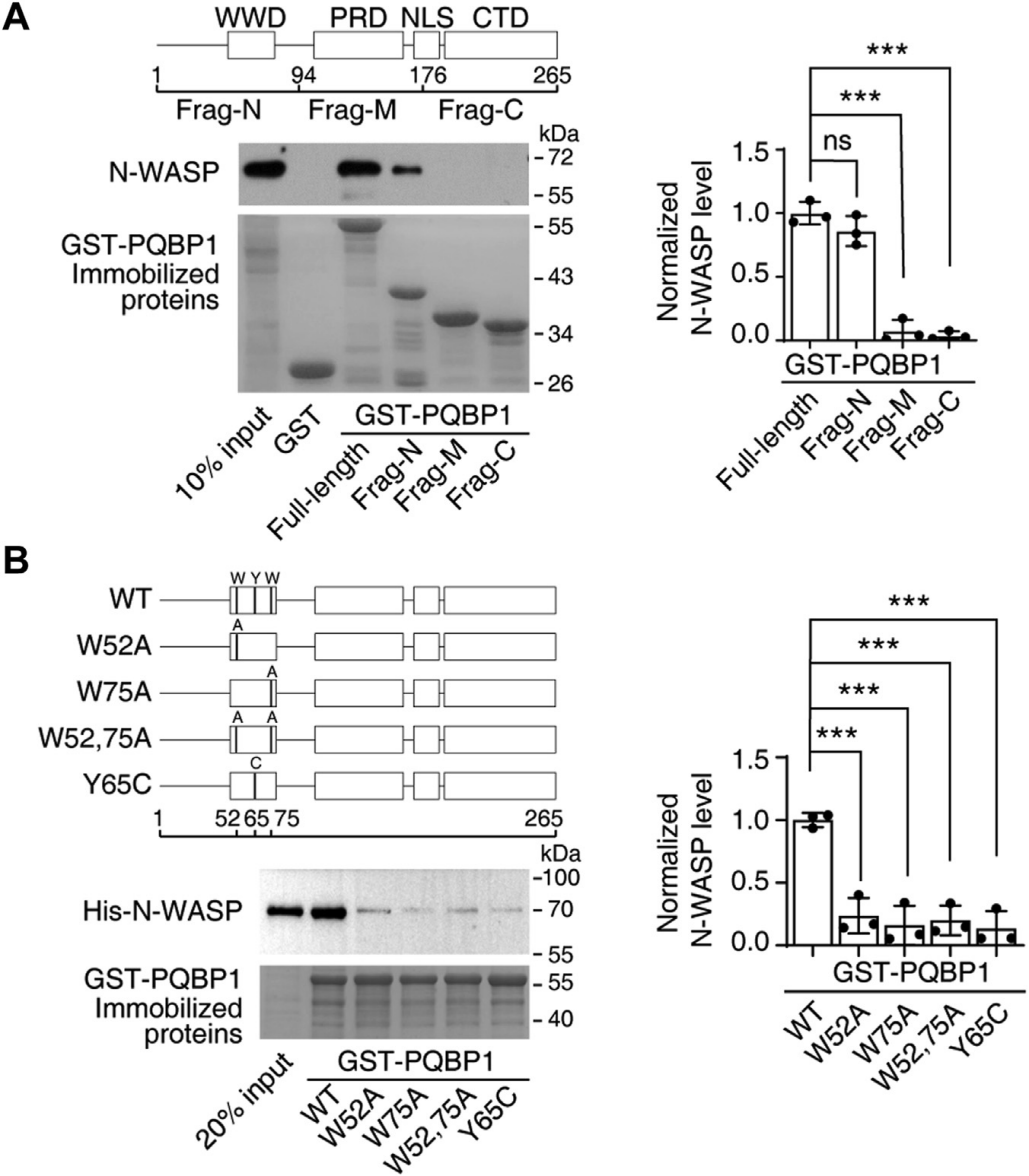
**PQBP1 interacts with N-WASP through its WW domain.***A, in vitro* binding assays were used to identify the binding site for PQBP1 on N-WASP. *Left panel*: *Top*, structural diagram of PQBP1; *middle*, western blotting for N-WASP; *bottom*, the various GST-PQBP1 fusion fragments used for the pull-down assay are stained with *Coomassie blue*. *Right panel*: Quantification of the relative amount of N-WASP bound to the GST-PQBP1 fusion fragments. Data are represented as means ± SD of three independent experiments. One-way ANOVA, F = 101.3, *p* < 0.0001. Dunnett *post hoc* test (full-length *versus* frag-N, M and C), *p* = 0.1912, < 0.0001 and < 0.0001, respectively. *B, in vitro* binding assays showing the binding preference of N-WASP for the PQBP1 variants. *Left panel*: *Top*, structural diagram of PQBP1 variants; *middle*, western blotting for N-WASP; *bottom*, the different PQBP1 variants used for the pull-down assay are stained with *Coomassie blue*. *Right panel*: Quantification of the relative amount of N-WASP bound to the PQBP1 variants. Data are presented as means ± SD of three independent experiments. One-way ANOVA, F = 24.99, *p* < 0.0001. Dunnett *post hoc* test (WT *versus* W52A, W75A, W52, 75A and Y65C), *p* < 0.0001, < 0.0001, < 0.0001, and < 0.0001. (∗∗∗ denotes *p* < 0.001, ns denotes not significant).

The mammalian PQBP1 protein contains an N-terminal WW domain, a polar amino acid-rich domain (PRD), a nuclear localization signal (NLS), and a C-terminal domain (CTD) (27). Accordingly, we next sought to identify the critical N-WASP-interacting region in PQBP1 by generating a series of functional domain truncation variants of PQBP1 containing an N-terminal GST tag. These truncated forms of PQBP1 were immobilized on glutathione-agarose beads and mixed with a mouse brain lysate for an *in vitro* binding assay, followed by Western blot analysis. We found that N-WASP was pulled down by full-length PQBP1 and a truncation variant containing the WW domain but not by variants containing only the PRD or CTD (Fig. 2*A*).

The WW domain is a module involved in mediating protein–protein interactions. A previous amino acid sequence alignment analysis revealed that the tryptophan residues at positions 52 and 75 (W52 and W75) are highly conserved across the entire family of human WW domains. Thus, we generated three mutated PQBP1 proteins, PQBP1^W52A^, PQBP1^W75A^, and PQBP1^W52, 75A^, and performed GST binding assays. The results showed that these mutations largely disrupted the PQBP1/N-WASP interaction (Fig. 2*B*). Given that the Y65C missense mutation in the WW domain of PQBP1 has been identified in patients with Renpenning syndrome (3, 4), we further examined whether this mutation affects PQBP1/N-WASP interaction. GST binding assay revealed that PQBP1^Y65C^ significantly impaired PQBP1/N-WASP interaction (Fig. 2*B*). These results demonstrated that PQBP1 physically interacts with N-WASP through its WW domain.

### The proline-rich domain of N-WASP mediates the direct interaction with PQBP1

**Figure 3:**
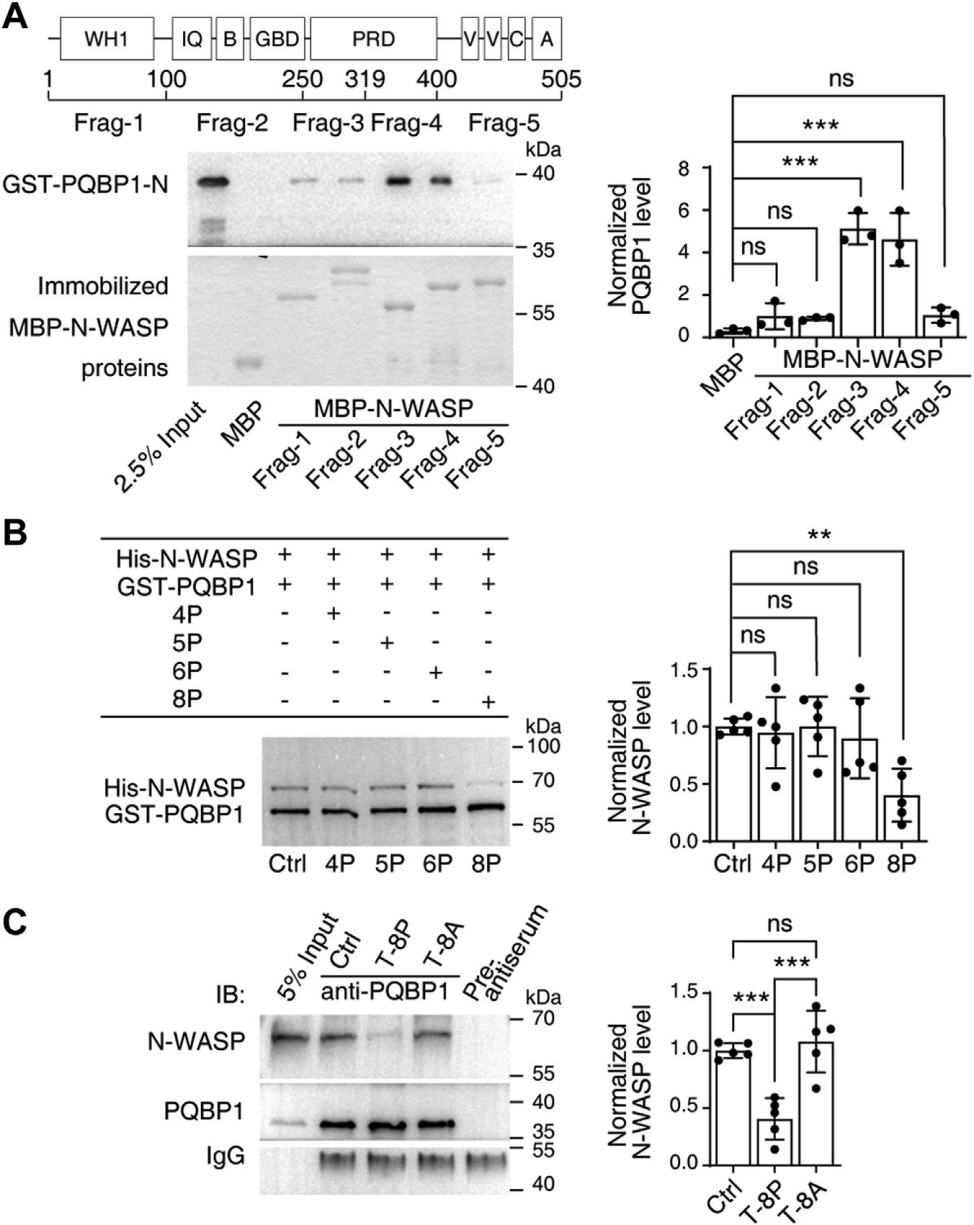
**The proline-rich domain of N-WASP mediates the association between N-WASP and PQBP1.***A, in vitro* binding assays showing the binding site for N-WASP on PQBP1. *Left panel*: *Top*, structural diagram of N-WASP; *middle*, western blotting for GST-PQBP1-N; *bottom*, the different MBP-N-WASP fusion fragments used for the pull-down assay are stained with *Coomassie blue*. *Right panel*: Quantification of the relative amount of GST-PQBP1-N bound to the MBP-N-WASP fusion fragments. Data are presented as means ± SD of three independent experiments. One-way ANOVA, F = 30.64, *p* < 0.0001. Dunnett *post hoc* test (MBP vs. Frag-1 to 5), *p* = 0.5834, 0.7144, < 0.0001, < 0.0001 and 0.5273, respectively. *B, in vitro* competition assays showing that the peptides with eight consecutive prolines reduce the binding between His-N-WASP and GST-PQBP1. *Upper panel*: *Coomassie blue* staining of His-N-WASP and GST-PQBP1. *Bottom panel*: Quantification of the relative amount of N-WASP bound to PQBP1. Data are represented as means ± SD of five independent experiments. One-way ANOVA, F = 4.677, *p* = 0.0079. Dunnett *post hoc* test (Ctrl *vs.* 4, 5, 6 and 8P), *p* = 0.9921, > 0.9999, 0.9255 and 0.0062, respectively. *C*, competition assays showing that membrane-permeable peptides with eight consecutive prolines reduce PQBP1/N-WASP interaction. *Left panel*: Co-immunoprecipitation of PQBP1 and N-WASP in neurons treated with membrane-permeable peptides with either eight consecutive prolines or eight consecutive alanines. *Right panel*: Quantification of the relative amount of N-WASP bound to PQBP1. Data are presented as means ± SD of five independent experiments. One-way ANOVA, F = 18.45, *p* = 0.0002. Tukey *post hoc* test (Ctrl *vs.* T-8P and T-8A), *p* = 0.0010 and 0.7964, and (T-8P *vs.* T-8A) *p* = 0.0003. (∗∗ denotes *p* < 0.01, ∗∗∗ denotes *p* < 0.001, ns denotes not significant).

As mentioned previously, N-WASP possesses a WH1 domain, a GBD, a PRD, and a G-actin-binding VCA domain (23). To determine which domain is involved in the interaction of N-WASP with PQBP1, we performed pull-down assays using several constructs harboring truncated N-WASP fragments fused to maltose-binding protein (MBP). The results revealed that the purified WW domain of PQBP1 bound strongly to the PRD of N-WASP but only weakly to the WH1, GBD, and VCA domains (Fig. 3*A*).

The WW domain of PQBP1 is a protein interaction motif, homologous to the SH3 domain that can recognize proline-rich regions of proteins (28, 29, 30). Given that N-WASP protein contains multiple poly-proline sequences (Fig. S1*A*), we next examined whether PQBP1 directly binds to N-WASP through a linear poly-proline epitope. To this end, we synthesized four poly-proline peptides containing four, five, six, and eight consecutive prolines, respectively (Fig. S1*B*), and performed an *in vitro* competitive binding assy. The results showed that the epitope with eight prolines had the highest efficiency in competing with PQBP1 for the interaction with His-N-WASP (Fig. 3*B*).

Next, we synthesized two membrane-permeable peptides containing either eight consecutive prolines or eight consecutive alanines. Co-immunoprecipitation results revealed that the membrane-permeable peptide with eight prolines was more effective than the membrane-permeable peptide with eight alanines at inhibiting the binding of N-WASP to PQBP1 (Fig. 3*C*). These data strongly indicated that PQBP1 binds directly to linear poly-proline motifs.

### PQBP1/N-WASP interaction is essential for neurite outgrowth in cultured hippocampal neurons

**Figure 4:**
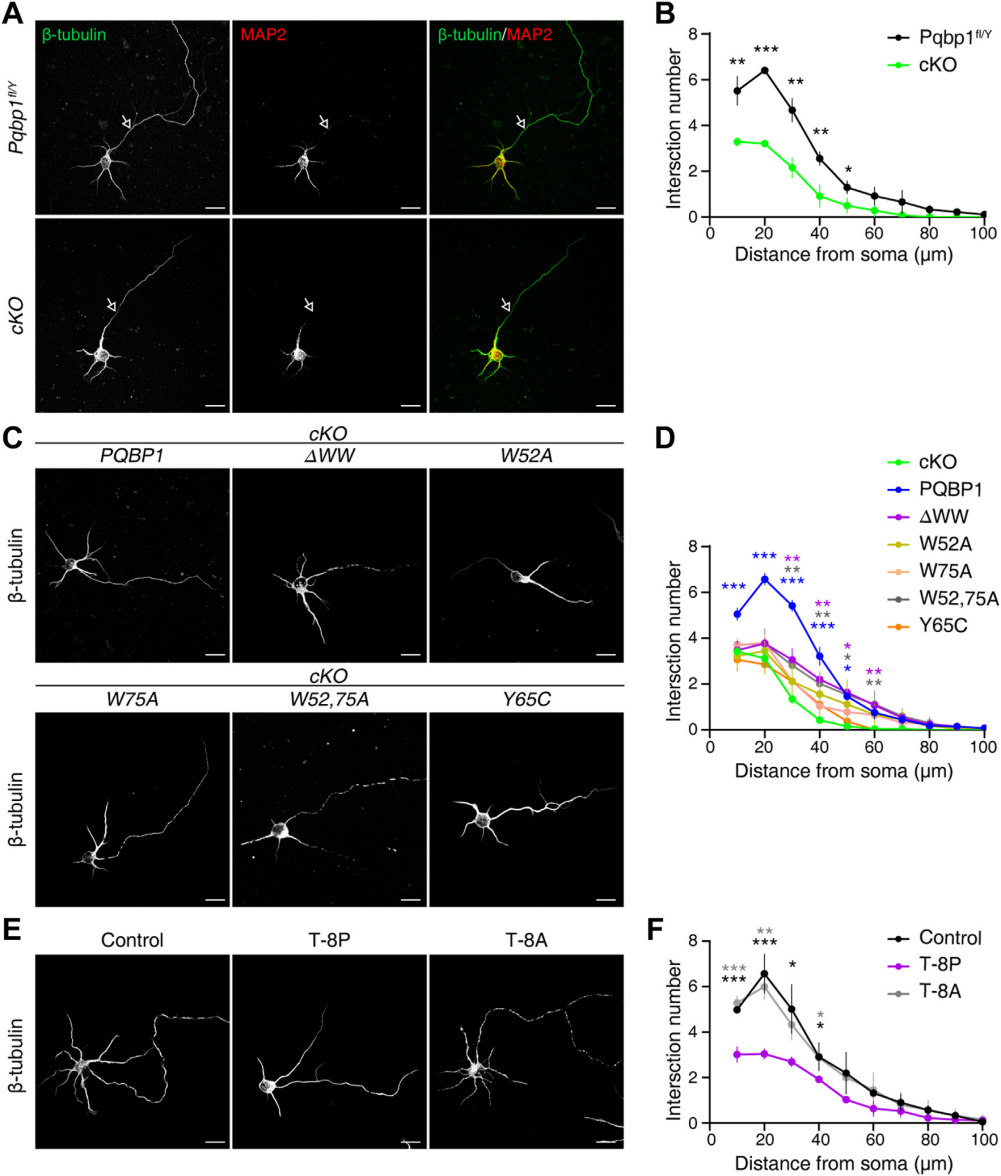
**PQBP1/N-WASP interaction is essential for neurite outgrowth in cultured hippocampal neurons.***A*, representative images of cultured *Pqbp1*^*fl/Y*^ and *Pqbp1* conditional knockout (cKO) hippocampal neurons were immunostained for MAP2 (*red*, a marker for neuronal dendrites) and β-tubulin (*green*, a marker for neurons) at 4 days *in vitro* (4 DIV). The arrow indicates the axon. Scale bar, 20 μm. *B*, sholl analysis revealed changes in neuronal dendrites complexity. ∗ represents significant difference between *Pqbp1*^*fl/Y*^ and cKO groups. Data are presented as means ± SD of three independent experiments. Two-tailed unpaired Student’s *t* test, (*Pqbp1*^*fl/Y*^*versus* cKO; 10–60 μm from soma), *p* = 0.0043, < 0.0001, 0.0030, 0.0083, 0.0334 and 0.0864, respectively. *C*, representative images of *Pqbp1*-cKO hippocampal neurons transfected with full-length PQBP1 or PQBP1 variants at 4 DIV. Scale bar, 20 μm. *D*, sholl analysis revealed changes in neuronal dendrites complexity. ∗ represents significant difference between rescue and cKO groups. The colors of the ∗ correspond to the color of the rescue groups. Data are presented as means ± SD of three independent experiments. One-way ANOVA and Dunnett *post hoc* test (cKO *versus* PQBP1, ΔWW, W52A, W75A, W52, 75 A and Y65C). 10 μm from soma, F = 10.46, *p* = 0.0002 (*p* = 0.0003, 0.9997, 0.9480, 0.8621, 0.8080 and 0.6550). 20 μm, F = 27.22, *p* < 0.0001 (*p* < 0.0001, = 0.2786, 0.8425, 0.2364, 0.2976 and 0.9144). 30 μm, F = 25.62, *p* < 0.0001 (*p* < 0.0001, = 0.0018, 0.2109, 0.2007, 0.0062 and 0.2109). 40 μm, F = 10.73, *p* = 0.0001 (*p* < 0.0001, = 0.0026, 0.0566, 0.4783, 0.0063 and 0.3676). 50 μm, F = 3.781, *p* = 0.0189 (*p* = 0.0384, 0.0178, 0.1662, 0.5226, 0.0280 and 0.9902). 60 μm, F = 5.592, *p* = 0.0038 (*p* = 0.0781, 0.0082, 0.1335, 0.1540, 0.0057 and 0.9998). *E*, representative images of wild-type, T-8P-treated and T-8A-treated hippocampal neurons at 4 DIV. Scale bar, 20 μm. *F*, Sholl analysis showed that dendrites complexity was decreased in T-8P-treated hippocampal neurons relative to that in wild-type or T-8A-treated neurons. ∗ represents significant difference between T-8P group and the other groups. The colors of the ∗ match the colors of each group. Data are represented as means ± SD of three independent experiments. One-way ANOVA and Dunnett *post hoc* test (T-8P *versus* Control and T-8A). 10 μm from soma, F = 55.93, *p* = 0.0001 (*p* = 0.0003 and 0.0001). 20 μm, F = 28.21, *p* = 0.0009 (*p* = 0.0008 and 0.0020). 30 μm, F = 7.420, *p* = 0.0239 (*p* = 0.0169 and 0.0661). 40 μm, F = 6.856, *p* = 0.0282 (*p* = 0.0298 and 0.0354). 50 μm, F = 2.632, *p* = 0.1511 (*p* = 0.1288 and 0.2070). 60 μm, F = 2.354, *p* = 0.1759 (*p* = 0.2357 and 0.1500). (∗ denotes *p* < 0.05, ∗∗ denotes *p* < 0.01, ∗∗∗ denotes *p* < 0.001, not significant not shown).

It has been shown that the knockdown of PQBP1 in mouse embryonic primary cortical neurons results in reduced dendrite branching and dendrite length (10). To further validate the roles of PQBP1 in neurite outgrowth, we generated *Pqbp1* conditional knockout (cKO) mice and assessed the morphology of primary hippocampal neurons isolated from these mice. Elaborate and branched neurites were observed in low-density cultured hippocampal neurons dissociated from *Pqbp1*^*fl/Y*^ (control) mice (Fig. 4*A*). In contrast, the depletion of PQBP1 led to a significant reduction in dendrite branching and dendrite length (Fig. 4, *A* and *B*). Sholl analysis further revealed that the complexity of the dendritic network was decreased in *Pqbp1-*depleted hippocampal neurons (Fig. 4*B*) (31).

N-WASP is a major cytoskeletal regulator, stimulating Arp2/3-mediated actin nucleation, which is critical for neurite outgrowth (14, 32, 33, 34, 35). To test whether PQBP1/N-WASP interaction is required for neurite outgrowth, we performed rescue experiments in cultured *Pqbp1-*depleted hippocampal neurons. The administration of full-length PQBP1, but not PQBP1^ΔWW^, which lacks the WW domain, reversed dendrite outgrowth impairment (Fig. 4*C*, *D*). Additionally, expressing the PQBP1 variants that disrupt PQBP1/N-WASP interaction (PQBP1^W52A^, PQBP1^W75A^, PQBP1^W52, 75A^, or PQBP1^Y65C^) failed to rescue the defects in neurite outgrowth in *Pqbp1*-cKO hippocampal neurons (Fig. 4, *C* and *D*). These results strongly indicated that PQBP1/N-WASP interaction is required for neurite outgrowth.

To further confirm that PQBP1/N-WASP interaction is required for neurite outgrowth, we examined the morphology of cultured wild-type hippocampal neurons following treatment with the membrane-permeable peptides harboring either eight consecutive prolines or eight consecutive alanines. We observed that the peptides containing eight consecutive prolines reduced dendrite branching and dendrite length in primary cultured hippocampal neurons, whereas those harboring eight consecutive alanines did not affect dendrite outgrowth (Fig. 4, *E* and *F*). We further elevated the specificity of the membrane-permeable peptides containing eight consecutive prolines on neurite outgrowth in primary cultured *Pqbp1-*cKO hippocampal neurons. No additive effect of the membrane-permeable peptides harboring eight consecutive prolines was observed on neurite outgrowth in *Pqbp1-*depleted hippocampal neurons (Fig. S2, *A* and *B*). These results provided further evidence that PQBP1/N-WASP interaction is required for neurite outgrowth.

### Disrupting of PQBP1/N-WASP interaction reduces growth cone size

**Figure 5:**
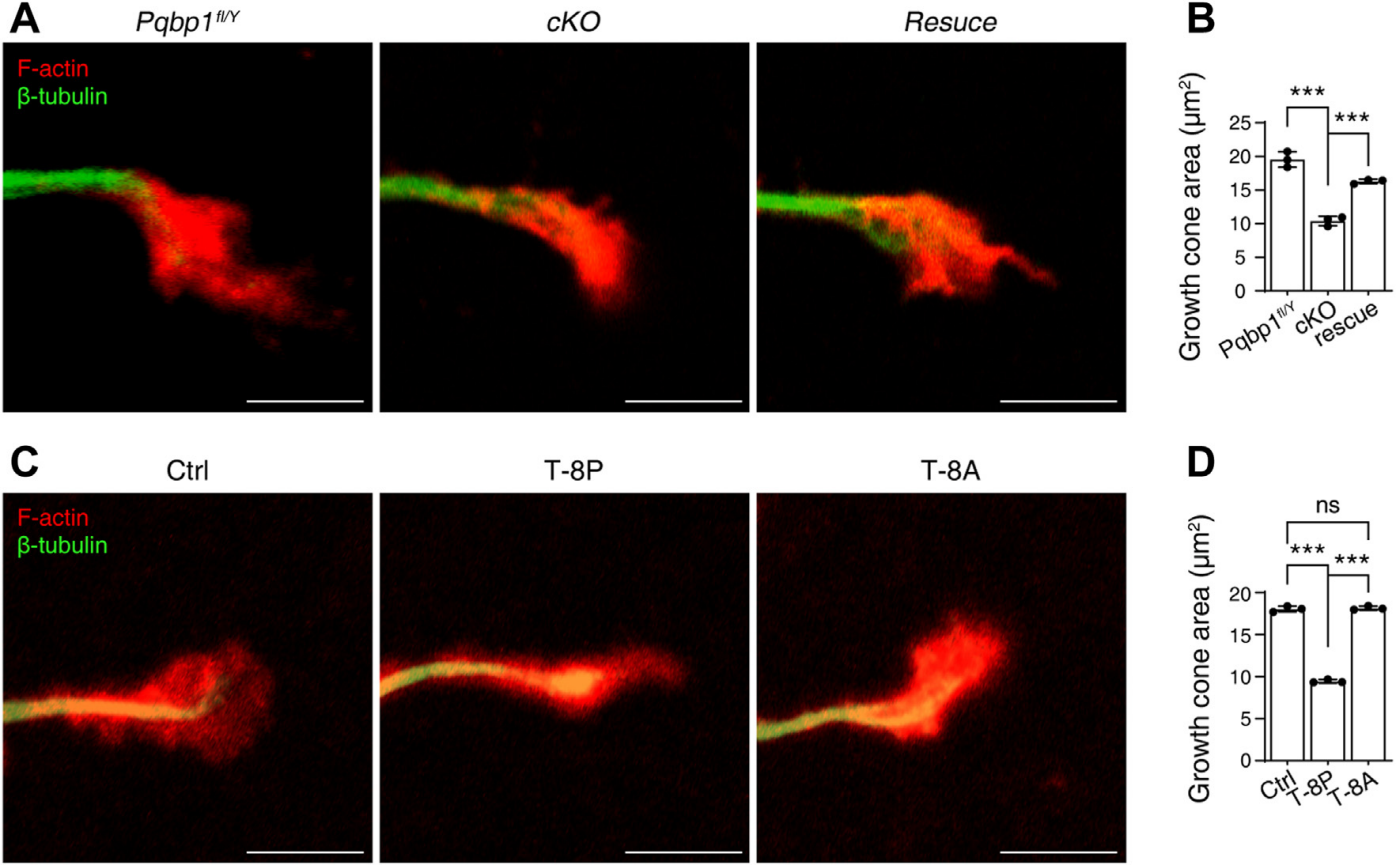
**The disruption of PQBP1/N-WASP interaction results in growth cone smaller.***A*, immunostaining image showing the morphology of the growth cones of *Pqbp1*^*fl/Y*^ and *Pqbp1* conditional knockout (cKO) hippocampal neurons as well as *Pqbp1*-cKO hippocampal neurons transfected with full-length Flag-hPQBP1 (rescue) at 2 days *in vitro* (DIV). Scale bar, 5 μm. *B*, quantification of the growth cone area in the indicated hippocampal neurons. Data are shown as means ± SD of three independent experiments. One-way ANOVA, F = 98.76, *p* < 0.0001. Dunnett *post hoc* test (cKO *versusPqbp1*^*fl/Y*^ and rescue), *p* < 0.0001 and *p* = 0.0002. *C*, immunostaining images showing the morphology of growth cones of wild-type hippocampal neurons treated with T-8P or T-8A peptides at 2 DIV. Scale bar, 5 μm. *D*, quantification of the growth cone area in T-8P and T-8A peptide-treated neurons. Data are presented as means ± SD of three independent experiments. One-way ANOVA, F = 1072, *p* < 0.0001. Tukey *post hoc* test (Ctrl *versus* T-8P and T-8A), *p* < 0.0001 and *p* = 0.8687, and (T-8P *vs.* T-8A) *p* < 0.0001. (∗∗∗ denotes *p* < 0.001, ns denotes not significant).

Neurite outgrowth during the initial stages of neuronal differentiation largely depends on the formation of the growth cone. Meanwhile, N-WASP-stimulated actin nucleation plays a critical role in growth cone expansion (13, 14, 32, 33, 36, 37). To determine whether PQBP1/N-WASP interaction regulates growth cone extension, we examined growth cone morphology in primary cultured *Pqbp1*-cKO hippocampal neurons. We found that the depletion of PQBP1 reduced growth cone size in these neurons (Fig. 5, *A* and *B*), and an effect that was reversed with the administration of full-length PQBP1 (Fig. 5, *A* and *B*). Similar results were obtained when primary cultured wild-type hippocampal neurons were treated with membrane-permeable peptides harboring eight consecutive prolines but not eight consecutive alanines (Fig. 5, *C* and *D*). These results demonstrated that PQBP1/N-WASP interaction promotes growth cone larger.

### PQBP1/N-WASP interaction does not affect the protein level of N-WASP, or induce N-WASP-mediated actin polymerization

**Figure 6:**
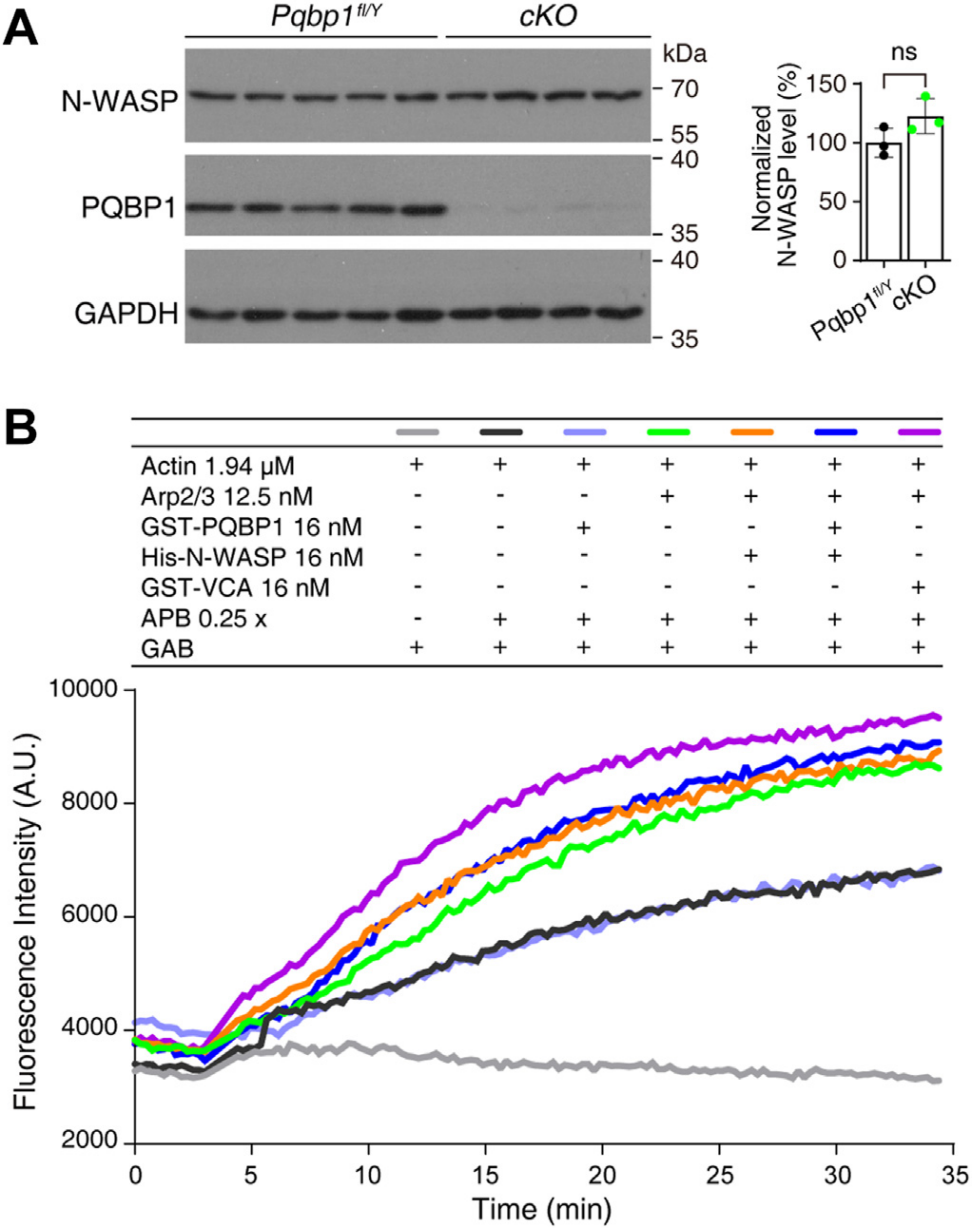
**PQBP1 does not affect the protein level of N-WASP or promote N-WASP-induced actin assembly.***A*, Western blots showing PQBP1 and N-WASP levels in conditional knockout (cKO) neurons. *Right panel*: Quantification of relative protein levels. Data are presented as means ± SD of three independent experiments. Two-tailed unpaired Student’s *t* test (*Pqbp1*^*fl/Y*^*versus* cKO), *p* = 0.1111. (ns denotes not significant). *B*, pyrene-actin polymerization assays showed that the addition of purified PQBP1 does not promote actin polymerization.

To investigate whether PQBP1/N-WASP interaction is required for N-WASP expression, we measured the protein level of N-WASP in the *Pqbp1*-depleted hippocampus by Western blot. The results showed that the level of N-WASP in *Pqbp1-*cKO hippocampal neurons was comparable to that in control hippocampal neurons (Fig. 6*A*), implying that PQBP1/N-WASP interaction is not required for N-WASP expression.

Given that N-WASP can stimulate Arp2/3-mediated actin nucleation (14, 33), we next assessed whether PQBP1/N-WASP interaction could enhance Arp2/3-mediated actin nucleation and, subsequently, also actin polymerization. We carried out an *in vitro* actin assembly assay to assess whether PQBP1 could promote N-WASP-induced actin polymerization. Consistent with previous reports, the activity of the Arp2/3 complex was partially enhanced when full-length N-WASP protein was administered and significantly enhanced by the administration of the VCA domain of N-WASP (Fig. 6*B*) (38, 39). In contrast, purified PQBP1 could not induce Arp2/3-mediated actin polymerization, either alone or in combination with N-WASP (Fig. 6*B*). These findings excluded the possibility that PQBP1/N-WASP interaction enhances Arp2/3-mediated actin nucleation and thus promote actin polymerization.

### PQBP1 recruits N-WASP to growth cone

**Figure 7:**
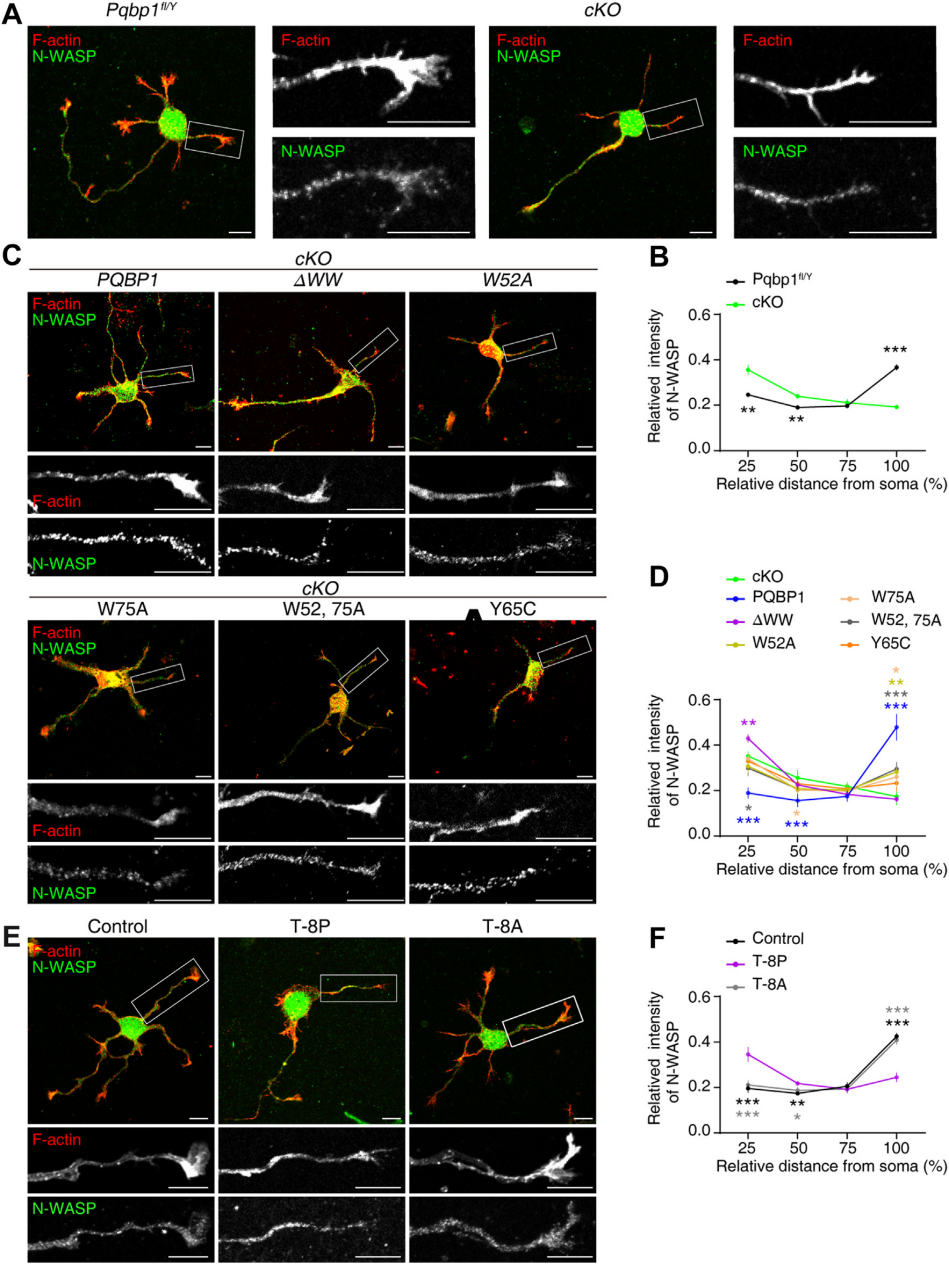
**PQBP1 recruits N-WASP to the growth cone.***A*, immunostaining images showing the distribution of N-WASP in cultured *Pqbp1*^*fl/Y*^ and *Pqbp1* conditional knockout (cKO) hippocampal neurons at 2 days *in vitro* (DIV). Scale bar, 10 μm. Higher magnification images of the white boxes are displayed right. Scale bar, 10 μm. *B*, quantification of the relative N-WASP intensity in neurites. ∗ represents a significant difference between *Pqbp1*^*fl/Y*^ and cKO groups. Two-tailed unpaired Student’s *t* test (*Pqbp1*^*fl/Y*^*vs.* cKO), 25% to 100% from soma, *p* = 0.0011, 0.0030, 0.1844 and < 0.0001, respectively. *C*, immunostaining images showing the distribution of N-WASP in cultured *Pqbp1*-cKO hippocampal neurons transfected with full-length PQBP1 or PQBP1 variants at 2 DIV. Higher magnification images of the white boxes are displayed below. Scale bar, 10 μm. *D*, quantification of the relative N-WASP intensity in neurites. ∗ represents significant difference between rescue and cKO groups. The colors of the ∗ correspond to the color of the rescue groups. One-way ANOVA and Dunnett *post hoc* test (cKO *vs.* PQBP1, ΔWW, W52A, W75A, W52, 75 A and Y65C). 25% from soma, F = 29.71, *p* < 0.0001 (*p* < 0.0001, = 0.0022, 0.1149, 0.9744, 0.0475 and 0.7077). 50%, F = 6.088, *p* = 0.0008 (*p* < 0.0001, = 0.3268, 0.0597, 0.0281, 0.0570 and 0.5056). 75%, F = 1.979, *p* = 0.1145. 100%, F = 35.74, *p* < 0.0001 (*p* < 0.0001, 0.9923, 0.0015, 0.0116, 0.0005 and 0.1184). *E*, immunostaining images showing the distribution of N-WASP in wild-type hippocampal neurons and neurons treated with the T-8P or T-8A peptides at 2 DIV. Higher magnification images of the white boxes are displayed below. Scale bar, 10 μm. *F*, quantification of the relative N-WASP intensity in the indicated neurons. ∗ Represents significant difference between T-8P group and the other groups. The colors of the ∗ match the colors of each group. Data are presented as means ± SD of three independent experiments. One-way ANOVA and Dunnett *post hoc* test (T-8P *vs.* control and T-8A). 25% from soma, F = 35.45, *p* = 0.0005 (*p* = 0.0005 and 0.0009). 50%, F = 16.56, *p* = 0.0036 (*p* = 0.0025 and 0.0133). 75%, F = 0.6871, *p* = 0.5387. 100%, F = 93.87, *p* < 0.0001 (*p* < 0.0001 and < 0.0001). (∗ denotes *p* < 0.05, ∗∗ denotes *p* < 0.01, ∗∗∗ denotes *p* < 0.001, not significant not shown).

To further explore the potential roles of PQBP1/N-WASP interaction in promoting growth cone size and neurite outgrowth, we next sought to determine whether disrupting this interaction affected the distribution of N-WASP. Immunostaining revealed that both N-WASP and PQBP1 distributed along neurites and were enriched in the growth cones of primary cultured control hippocampal neurons (Figs. 7, *A* and *B* and S3, *A* and *C*). In contrast, *Pqbp1-*cKO hippocampal neurons distributed along neurites, but exhibited significantly reduced N-WASP distribution in growth cone (Figs. 7, *A* and *B* and S3*A*). The expression of full-length PQBP1 but not its truncated (PQBP1^ΔWW^) or mutated PQBP1 (PQBP1^W52A^, PQBP1^W75A^, and PQBP1^W52, 75A^) forms, restored correct N-WASP distribution in the growth cones in *Pqbp1*-cKO hippocampal neurons (Fig. 7, *C* and *D*). Similarly, PQBP1^Y65C^ also failed to reverse the changes in N-WASP distribution in the growth cone observed in *Pqbp1*-depleted hippocampal neurons (Fig. 7, *C* and *D*). In contrast, the expression of different PQBP1 forms, restored PQBP1 enriched distribution in the growth cones in *Pqbp1*-cKO hippocampal neurons (Fig. S3, *B* and *C*). These data indicated that PQBP1/N-WASP interaction is unnecessary for adjusting PQBP1 positioning, and is required for recruiting N-WASP to the growth cone.

We also investigated whether disrupting PQBP1/N-WASP interaction through the provision of membrane-permeable peptide with eight consecutive prolines could change the distribution of N-WASP in the growth cone. We found that the administration of the membrane-permeable peptide did not affect the distribution of PQBP1 (Fig. S3, *D* and *E*), but significantly reduced N-WASP distribution in the growth cone (Fig. 7, *E* and *F*). These results demonstrated that PQBP1/N-WASP interaction promotes the recruitment of N-WASP to the growth cone.

## Discussion

### PQBP1 directly interacts with N-WASP

Vertebrate PQBP1 is a nucleocytoplasmic shuttling protein with roles in transcription, mRNA splicing, and translation (10, 12, 27, 40, 41, 42). PQBP1 contains a WW domain, a polar amino acid-rich domain, a NLS, and a CTD (40, 43). In our recent work, using a combination of co-immunoprecipitation and LC-MS/MS analyses, we identified 160 potential PQBP1-interacting proteins, with N-WASP being one of the top hits (9). Although PQBP1 and N-WASP are expressed ubiquitously, the highest abundance of both proteins is found in the brain (22, 23, 40, 44). In this study, our immunostaining and co-immunoprecipitation assays confirmed that PQBP1 physically associates with N-WASP *in vivo*. Moreover, we found that PQBP1 directly interacts with N-WASP through its N-terminal WW domain. Studies have shown that the WW domain specifically recognizes six groups of proline-rich motifs and binds the PPxPP motif (28, 45).

N-WASP is a critical regulator of actin dynamics, promoting actin polymerization by triggering Arp2/3-mediated actin nucleation (16, 17, 18, 19, 20, 21). N-WASP possesses multiple functional domains (22, 23, 24) and our *in vitro* binding and competition assays revealed that the Pro (PRD) domain of N-WASP mediates the interaction between PQBP1 and N-WASP. It has been reported that several SH3 domain-containing proteins, such as WISH, GRB2, NCK, and Toca-1, interact with the Pro domain of N-WASP, thereby activating it (46, 47, 48, 49). The WW domain structurally resembles the SH3 domain and recognizes proline-rich motifs in proteins (50). Relevant to our study, it has been demonstrated that Gas7 and FBP11 interact directly with N-WASP *via* their WW domains (34, 51).

### PQBP1 recruits N-WASP to the growth cone which facilitates neurite outgrowth

Mutations in the *PQBP1* gene have been associated with Renpenning syndrome. Additionally, studies in animal models and cultured cells have shown that PQBP1 plays essential roles in neural stem progenitor cell proliferation and differentiation as well as neurite outgrowth (6, 7, 8, 9, 10, 11). In this work, we found that *Pqbp1*-depleted mouse hippocampal neurons exhibited significantly reduced dendrite branching and length. This result is consistent with the observation that in the knockdown of PQBP1 in neurons substantially diminished dendrite branching and length (10). These findings demonstrate that PQBP1 is essential for neurite outgrowth.

Neurite outgrowth begins with protrusions of the neuronal membrane and depends largely on the formation of growth cones, which sense internal and external cues, and guide the continuous addition and retraction of dendritic branches (52). N-WASP-stimulated actin nucleation is critical for growth cone expansion (13, 14, 32, 33, 36, 37). Here, we demonstrated that PQBP1/N-WASP interaction is essential for neurite outgrowth, and the disruption of this interaction resulted in growth cone smaller.

Following its activation by Cdc42 and phosphatidylinositol 4,5-bisphosphate, N-WASP recruits the Arp2/3 complex, which initiates actin polymerization (16, 24, 53). Several proteins have been shown to activate quiescent N-WASP by binding to its Pro region (46, 47, 48, 49). These proteins cooperate with GTP-Cdc42/phosphatidylinositol 4,5-bisphosphate to fully activate N-WASP. However, our *in vitro* actin assembly assay demonstrated that PQBP1/N-WASP interaction does not stimulate actin polymerization; instead, we found that disrupting PQBP1/N-WASP interaction reduced N-WASP abundance in the growth cone. Our study indicated that direct PQBP1/N-WASP interaction plays a significant role in neurite outgrowth and growth cone size. This represents an alternative molecular mechanism by which PQBP1 mediates neurite outgrowth and an alternative explanation for the mechanism underlying the pathogenesis of Renpenning syndrome. However, cannot exclude the possibility that PQBP1-mediated transcription, mRNA splicing, and translation may also be involved in the regulation of neurite outgrowth.

## Experimental procedures

### Antibodies and reagents

Anti-PQBP1 polyclonal and monoclonal antibodies were raised in rabbits against full-length GST-PQBP1 and His-PQBP1, respectively (12). Anti-PQBP1-C monoclonal antibody was raised in rabbits against GST-PQBP1-C (189-263 aa) fusion protein by YUROGEN. The specificity of anti-PQBP1-C antibody for immunostaining was verified in *Pqbp1*^*fl/Y*^ and *Pqbp1* cKO hippocampal neurons. The primary antibodies used in this study were rabbit monoclonal anti-N-WASP antibody (Cat: 4848, Cell Signaling Technology), mouse monoclonal anti-N-WASP antibody (Cat: sc-271484), mouse monoclonal anti-MAP2 antibody (Cat: M4403, Sigma-Aldrich), rabbit polyclonal anti-β-tubulin antibody (Cat: ab6046, Abcam), mouse monoclonal anti-GST antibody (Cat: M20007, Abmart), rabbit polyclonal anti- His-tag antibody (Cat: AE068, ABclonal), and mouse monoclonal anti-GAPDH antibody (Cat: MA5-15738, Thermo Fisher). Phalloidin-iFluor 647 Reagent (Cat: ab176759) was purchased from Abcam. Alexa Fluor 488 goat anti-rabbit IgG (Cat: ab150081), Alexa Fluor 555 anti-rabbit IgG (Cat: ab150078), Alexa Fluor 488 anti-mouse IgG (Cat: ab150117), and Alexa Fluor 555 anti-mouse IgG (Cat: ab150118) antibodies were obtained from Abcam. Horseradish peroxidase (HRP)-conjugated goat anti-mouse IgG (Cat: 401215) and goat anti-rabbit IgG (Cat: 401315) antibodies were obtained from Millipore.

Glutathione-Sepharose resin (Cat: 786-311) was purchased from GE Healthcare. Amylose resin (Cat: E8021S) was purchased from New England Biolab (NEB). Protein A beads (Cat: 20333) were obtained from Thermo Fisher. Ni-NTA agarose (Cat: 30210) was from QIAGEN (GER). Actin protein (Cat: CSK-AKL95-B), pyrene-actin protein (Cat: AP05-A), and the Arp2/3 protein complex (Cat: RP01P-A) were obtained from Cytoskeleton, Inc.

## Animals

*Pqbp1*^*fl/fl*^ mice were generated as previously described (12). *Pqbp1* cKO mice were produced by crossing *Nestin-Cre* mice with *Pqbp1*^*fl/fl*^ mice. *Pqbp1*^*fl/Y*^ male littermates were used as controls. C57BL/6 mice were purchased from Qinglong Mountain animal breeding farm. All animal experiments were approved by the Institutional Animal Care and Use Committee of Southeast University. The mice were maintained in a barrier facility at 25 °C under a regular 12-h light/12-h dark cycle.

### Plasmid construction

cDNA encoding the full-length N-WASP protein was inserted into the *pMALTEV*, *pET15B*, or *pFastBacHTA* vectors. N-WASP fragments (Frag-1, 1–100 aa; Frag-2, 101–250 aa; Frag-3, 251–319 aa; Frag-4, 320–400 aa; Frag-5, 401–505 aa) were inserted into *pMALTEV* vector. The plasmids *pGEXTEV-hPQBP1*, *pGEXTEV-hPQBP1-N*, *pGEXTEV-hPQBP1-M*, *pGEXTEV-hPQBP1-C*, and *pFlag-CMV2-hPQBP1* were generated in our previous study (8). A list of the constructs and primers used in this study is provided in Table S1.

### Recombinant protein expression

All MBP and GST fusion proteins were expressed in *E. coli* BL21 cells. The cells were harvested by centrifugation and lysed in ice-cold column buffer (20 mM Tris-HCl pH 7.4, 200 mM NaCl, 1 mM EDTA, 1 mM DTT) containing 1% w/v protease inhibitor or in 1 × PBS containing 1% Triton X-100 and 1% w/v protease inhibitor. His-N-WASP proteins were expressed in *E. coli* BL21 cells and Baculovirus/Sf9 cells, which were obtained from Jiangsu Cowin Biotech Co, Ltd (CWBIO) and Beijing Biobw Biotechnology Co, Ltd (Biobw), for competitive binding and pyrene-actin polymerization assays. The cells were harvested by centrifugation and lysed in ice-cold buffer A (20 mM Tris-HCl pH 8.0, 500 mM NaCl, 20 mM imidazole, 5% glycerin, 2 mM β-mercaptoethanol). The resulting lysates were purified by affinity chromatography. Eluates were concentrated and stored at −80 °C for further analysis.

### Co-immunoprecipitation assays

Cytoplasmic proteins obtained from the brains of 8-10-day-old C57BL/6 mice were incubated first with 2 μl of pre-immunized rabbit serum or anti-PQBP1 polyclonal antibody for 1 h at 4 °C, and then with 150 μl of protein A beads for 30 min at 4 °C. After three washes, the immune complexes were eluted with 2 × SDS sample buffer and loaded onto SDS polyacrylamide gels for Western blot.

Hippocampal neurons isolated from 1-day-old C57BL/6 mice were lysed and then incubated with T-8P or T-8A peptides (4 μg per mouse) for 2 h. The lysate was incubated with 2 μl of anti-PQBP1 polyclonal antibody and then with 150 μl of protein A beads for 30 min at 4 °C. After washing three times, the immune complexes were eluted with 2 × SDS sample buffer and loaded onto SDS–polyacrylamide gels for Western blot.

### Microscale thermophoresis

The His-Tag Labeling Kit RED-tris-NTA second Generation (Cat: MO-L018, NanoTemper, GER) was used to measure the binding affinity between PQBP1 and N-WASP. One hundred microliters of dye (100 nM) was added to 100 μl of His-N-WASP protein (400 nM), mixed, and left to stand for 30 min away from light. The supernatant was harvested by centrifugation at 15,000 rpm for 10 min at 4 °C. PQBP1 protein, diluted with PBS-T buffer (Phosphate buffer saline with 0.01% Tween 20), was transferred to PCR tubes No. 1-16, and His-N-WASP protein was then added to the tubes, followed by mixing. The working concentration of His-N-WASP protein was 100 nM. The maximum working concentration of PQBP1 protein was 10 μM. The sample was absorbed in a capillary tube (Cat: MO-K022, NanoTemper) and tested by micro-thermophoretic molecular interaction analyzer (Monolith NT.115).

### Pull-down assays

To map the N-WASP-interacting site in PQBP1, GSH resins were coupled to GST-PQBP1 fusion proteins (Full-length, 1–265 aa; Frag-N, 1–94 aa; Frag-M, 95–176 aa; Frag-C, 177–265 aa) for 2 h at 4 °C, and then incubated with SK-N-BE (2) cell, which was obtained from Shanghai Institute of Biochemistry and Cell Biology (SIBCB), at 4 °C overnight. To map the PQBP1-binding site in N-WASP, MBP-N-WASP fusion proteins (Frags 1–5) were coupled to amylose resin for 2 h, and then the amylose resin was incubated with GST-PQBP1-Frag-N proteins overnight at 4 °C. After three washes with 0.1% PBST (Phosphate buffer saline with 0.1% Triton X-100), the resin was incubated with 2 × SDS sample buffer, boiled and subjected to SDS-PAGE gels for Western blot.

To test the binding preference of N-WASP for PQBP1 variants, the GST fusion PQBP1 variant (5.8 μg) were incubated with GSH resin (20 μl) in 1 ml PBS for 2 h at 4 °C. After three washes with 0.1% PBST, the GSH resin coated with the GST-PQBP1 variants was incubated with purified His-N-WASP protein (7.0 μg) in PBS for 2 h at 4 °C. After extensive washing, the bound N-WASP proteins were analyzed by Western blot.

### Peptide preparation

The peptides used in this study were synthesized and purified by GenScript (China) (purity: 95%). The peptide sequences were as follows: 4P, PPPPAAAP; 5P, PPPPPAAP; 6P, PPPPPPAP; 8P, PPPPPPPP; T-8A, GRKKRRQRRRPPQ-AAAAAAAA; and T-8P, GRKKRRQRRRPPQ-PPPPPPPP. The peptides were resuspended in PBS to a concentration of 1 mg/ml and stored at −20 °C.

### Competitive binding assay

GST-PQBP1 protein (5.8 μg per reaction) immobilized on GSH resins was incubated with peptides (4P, 5P, 6P, or 8P; 30 μg per reaction) for 2 h at 4 °C. The same volume of PBS served as the ctrl. After washing three times with 0.1% PBST, the GSH resin coated with GST-PQBP1 protein was incubated with His-N-WASP protein (7.0 μg per reaction) for 2 h at 4 °C. After extensive washing, the bound N-WASP proteins were analyzed by Western blot.

### Primary neuronal culture and peptide treatment

Cultures of primary hippocampal neurons were performed as previously described (8, 54). The hippocampus was dissected and dissociated using ice-cold PBS (Cat: 311-011-CL, Wisent, CAN) containing 1% penicillin/streptomycin (Cat: 450-201-CL, Wisent). Tissue fragments were trypsinized (Trypsin Cat: 25300120) at 37 °C for 15 min and subjected to mechanical dissociation by repeated aspirations through a Pasteur pipette. Hippocampal neurons isolated from one mouse were equally separated into eight wells of a 24-well plate and plated onto poly-D-lysine (100 μg/ml)-coated glass coverslips. The primary hippocampal neurons were cultured in neurobasal A medium (Cat: 10888022, Gibco) supplemented with B-27 (Cat: 17504044, Gibco), GlutaMAX (Cat: 35050061, Gibco), and penicillin/streptomycin in an incubator under 5% CO_2_ at 37 °C.

Half-medium changes and peptide treatment (T-8A or T-8P, 0.5 μg/well, 1 μg/ml) were performed after 2 and 24 h. Growth cone morphology and neurite growth were analyzed at 2 days *in vitro* (DIV) and 4 DIV, respectively.

### Pyrene-actin polymerization

The pyrene-actin polymerization assay was performed with the Actin Polymerization Biochem kit (catalog no. BK003, Cytoskeleton, Inc) according to the manufacturer’s instructions. For each assay, the proteins (Arp2/3 at 12.5 nM, GST-PQBP1 at 16 nM, His-N-WASP at 16 nM, and GST-VCA at 16 nM) tested were combined with 0.25 × actin polymerization buffer (12.5 mM KCl, 0.5 mM MgCl_2_, 2.5 mM Tris-HCl pH = 7.5, 0.25 mM ATP) and diluted in general actin buffer (5 mM Tris-HCl pH = 8.0, 0.2 mM CaCl_2_). Globular actin (G-actin, 0.125 mg/ml) was prepared by mixing pyrene-labeled muscle actin (20%) with unlabeled muscle actin in general actin buffer containing 0.2 mM ATP, and then incubated for 1 h on ice. After centrifugation at 15,000 rpm for 30 min at 4 °C, the supernatant was harvested as G-actin solution. The 60 μl G-actin solution were transferred into a 96-well black plate, and the baseline fluorescence at 410 nm (excitation: 350 nm) was immediately monitored in a microplate reader (BioTek Gen5) every 15 s for 3 min. Then, 30 μl protein mixture were added in, shaken for 5 s, and the increase in fluorescence was monitored every 15 s for 30 min. Each reaction system has a volume of 90 μl.

### Cell transfection

Hippocampal neurons isolated from *Pqbp1-*cKO mice were plated onto a 24-well culture dish. After 24 h, the neurons were transfected with the plasmids (0.5 μg/well) *pFlag-CMV2-hPQBP1*, *pFlag-CMV2-hPQBP1*^*ΔWW*^*, pFlag-CMV2-hPQBP1*^*W52A*^*, pFlag-CMV2-hPQBP1*^*W75A*^*, pFlag-CMV2-hPQBP1*^*W52, 75A*^*,* and *pFlag-CMV2-hPQBP1*^*Y65C*^ using X-tremeGENE HP DNA transfection reagent (Cat: 06366236001, Roche) according to the manufacturer’s protocol. Growth cone morphology and neurite growth were analyzed at 2 and 4 DIV, respectively.

### Immunostaining and image acquisition

Cells were washed with PBS and fixed in 4% paraformaldehyde for 25 min at 37 °C, and then permeabilized with 0.5% PBST (Phosphate buffer saline with 0.5% Triton X-100) for 15 min at room temperature. After blocking in 10% fetal bovine serum (FBS, diluted in 0.1% PBST) at room temperature for 1 h, the cells were incubated first with primary antibodies overnight at 4 °C and then with the respective secondary antibodies at room temperature for 2 h. Filamentous actin (F-actin) was labeled with phalloidin at room temperature for 2 h. Images were captured with a Zeiss LSM 700 confocal laser scanning microscope.

### Statistical analysis

For Western blot analysis, the gray ratio between target strip and loading (or internal parameter) was analyzed and normalized (Figs. 2, 3 and and 6). For neuron morphology analysis and quantification of N-WASP distribution, three independent experiments were performed (Figs. 4, 5 and and 7), with more than eight neurons being analyzed in each experiment. The mean values with SD (as error bars) presented are the averages of three sets of data. For quantitative analysis of N-WASP distribution in neurites, the selected branches were evenly divided into four segments, and the relative N-WASP intensity was determined by comparing the signal of each segment with that of the selected branch.

Western blot and fluorescence images were analyzed with ImageJ software (National Institute of Health). Data analysis was performed using GraphPad Prism 8.0. Quantitative data are presented as means ± SD. One-way ANOVA (followed by Dunnett or Tukey *post hoc* test) or a two-tailed unpaired Student’s *t* test was used, as appropriate.

## Data availability

The data supporting the findings of this study are included in the paper and its supporting information. Further inquiries can be directed to the corresponding author.

## Supporting information

This article contains supporting information (8, 42).

## Conflict of interest

The authors declare that they have no conflicts of interest with the contents of this article.
