# Supplementary material for: Polyglutamine binding protein 1 regulates neurite outgrowth through recruiting N-WASP
Source: J Biol Chem. 2024 Jul 4;300(8):107537. doi: 10.1016/j.jbc.2024.107537 (PMC11339035; doi:10.1016/j.jbc.2024.107537)
Supplement: Supporting information [file mmc1.pdf]

Supporting information.

Polyglutamine Binding Protein 1 regulates neurite outgrowth through recruiting N-WASP

Xuejiao Huang, Shanshan Cheng, and Junhai Han

The list of the Supporting information included:

Supplementary Figure S1 (Related Figure 3). The proline-rich domain of N-WASP contains a consecutive poly-proline epitope.

Supplementary Figure S2 (Related Figure 4). The T-8P peptide does not affect neurite outgrowth in cultured *Pqbp1* conditional knockout (cKO) hippocampal neurons.

Supplementary Figure S3 (Related Figure 7). PQBP1/N-WASP interaction is not essential for the distribution of PQBP1.

Supplementary Table S1. Lists of vectors and primers used in this paper.

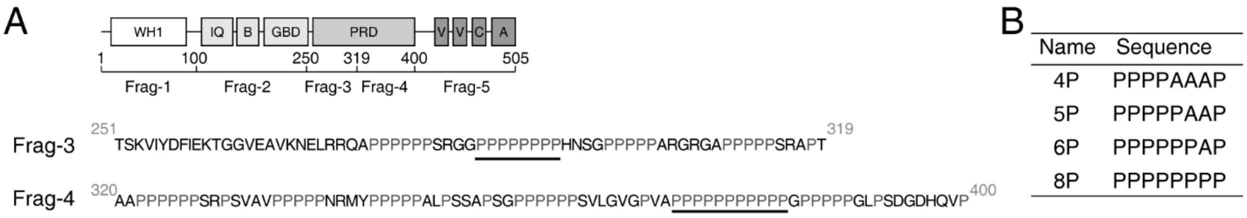

Supplementary Figure S1 (Related Figure 3). The proline-rich domain of N-WASP contains a consecutive poly-proline epitope. *A*, *Upper panel*: Structural diagram of N-WASP. *Bottom panel*: Amino acid sequence information of Frag-3 (251–319 aa) and Frag-4 (320–400 aa). Prolines are highlighted in gray. Two sites containing the epitope with eight consecutive prolines are marked with black bold lines. *B*, The sequences of the poly-proline peptides used for the competitive binding assay.

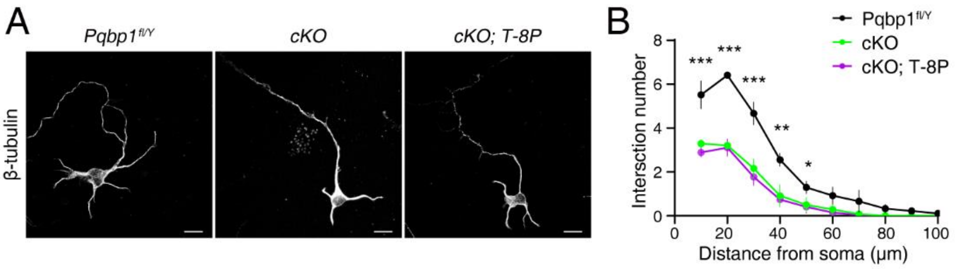

Supplementary Figure S2 (Related Figure 4). The T-8P peptide does not affect neurite outgrowth in cultured *Pqbp1* conditional knockout (cKO) hippocampal neurons. *A,* Representative images of *Pqbp1*$^{fl/Y}$ and *Pqbp1*-cKO hippocampal neurons as well as *Pqbp1*-cKO hippocampal neurons treated with the T-8P peptides (0.5 µg per well) at 4 days *in vitro*. Scale bar, 20 µm. *B,* Sholl analysis showed the changes in dendrite complexity. * represents significant difference between *Pqbp1*$^{fl/Y}$ and cKO groups. The difference between cKO and cKO; T-8P groups were not significant and were not marked. Data are presented as means ± SD of three independent experiments. One-way ANOVA and Dunnett *post hoc* test (cKO *vs. Pqbp1*$^{fl/Y}$ and cKO; T-8P). 10 µm from soma, F = 38.20, p = 0.0004 (p = 0.0008 and 0.4091). 20 µm, F = 170.7, p < 0.0001 (p < 0.0001 and = 0.8515). 30 µm, F = 36.86, p < 0.0001 (p = 0.0009 and 0.5016). 40 µm, F = 20.28, p = 0.0021 (p = 0.0036 and 0.8057). 50 µm, F = 8.087, p = 0.0198 (p = 0.0297 and 0.9017). 60 µm, F = 6.432, p = 0.0322 (p = 0.0580 and 0.7688). (* denotes p < 0.05, ** denotes p < 0.01, *** denotes p < 0.001, not significant not shown).

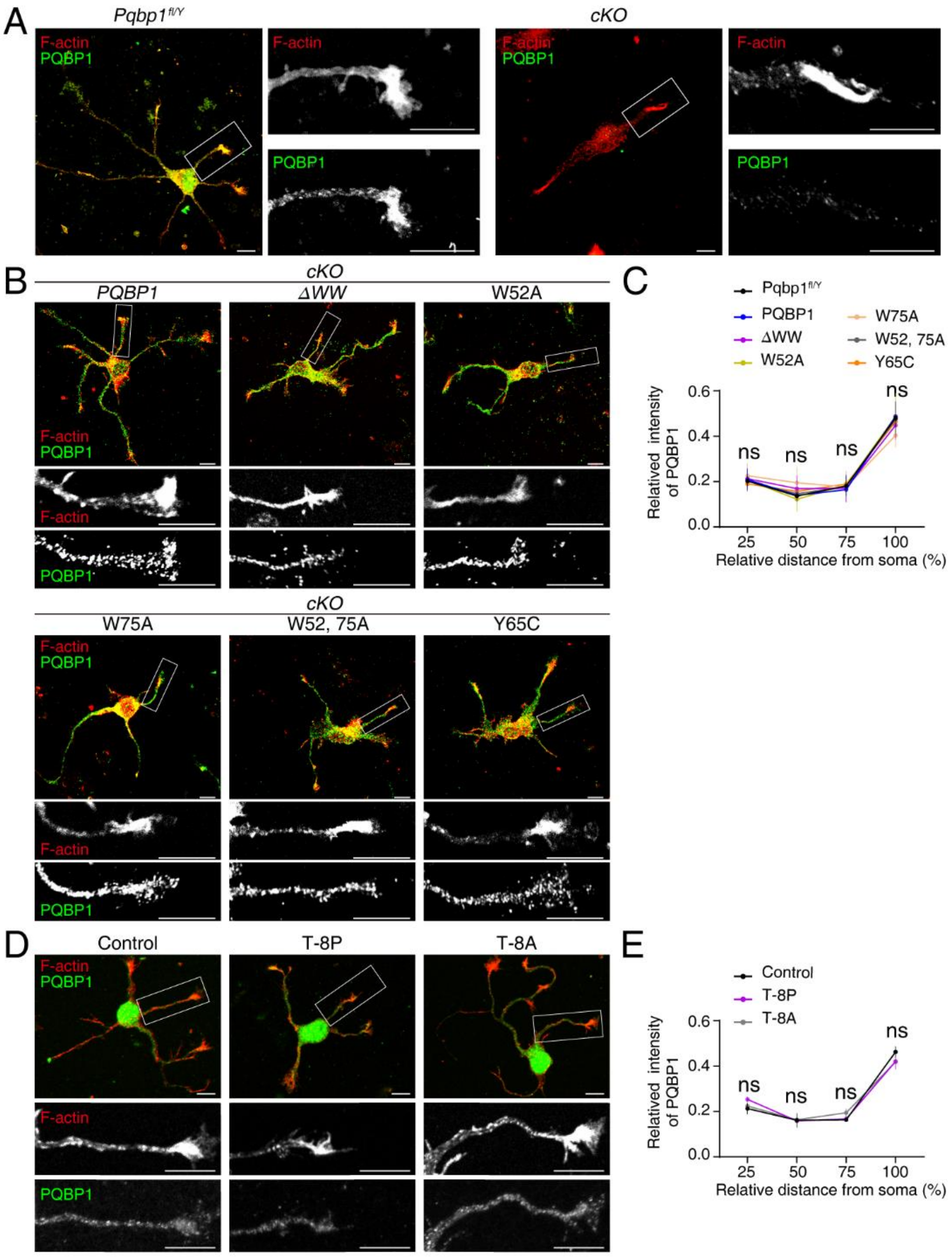

Supplementary Figure S3 (Related Figure 7). PQBP1/N-WASP interaction is not essential for the distribution of PQBP1. *A,* Immunostaining images showing the distribution of PQBP1 in *Pqbp1*$^{fl/Y}$ and *Pqbp1* conditional knockout (cKO) hippocampal neurons at 2 days *in vitro* (DIV). Scale bar, 10 µm. Higher magnification images of the white boxes are displayed right. *B,* Immunostaining images showing the distribution of PQBP1 in *Pqbp1*-cKO hippocampal neurons transfected with full-length PQBP1, truncated Flag-PQBP1$^{\Delta WW}$, and mutated PQBP1 proteins, PQBP1^{W52A}, PQBP1^{W75A}, PQBP1$^{W52, 75A}$, and PQBP1^{Y65C} at 2 DIV. Higher magnification images of the white boxes is displayed below (2 DIV). Scale bar, 10 µm. *C,* Quantification of the relative PQBP1 intensity in neurites. Each different salvage group was compared with the *Pqbp1*$^{fl/Y}$ group. One-way ANOVA, relative distance from soma, 25%, F = 0.2541, p = 0.9494. 50%, F = 0.7465, p = 0.6221. 75%, F = 0.09535, p = 0.9957. 100%, F = 0.4180, p = 0.8550. *D,* Immunostaining images showing the distribution of PQBP1 in wild-type hippocampal neurons as well as neurons treated with the T-8P or T-8A peptides (2 DIV). Scale bar, 10 µm *E,* Quantification of the relative PQBP1 intensity in the indicated neurons. Each different group was compared with the T-8P group. Data are presented as means ± SD of three independent experiments.

One-way ANOVA and Dunnett *post hoc* test (T-8P *vs.* Control and T-8A). Relative distance from soma, 25%, F = 4.760, $p = 0.0578$. 50%, F = 0.02673, $p = 0.9737$. 75%, F = 5.459, $p = 0.0446$ ($p = 0.8942$ and 0.0665). 100%, F = 3.066, $p = 0.1210$. (ns denotes not significant).

Supplementary Table S1. Lists of vectors and primers used in this paper.

Name	From	Vector	restriction Enzyme site	PCR-primer (5’ to 3’)
pMALTEV-hN-WASP	cloned	*pMALTEV*	*Xma*I/*Hind*III	F: ATTCCCGGGTTATGAGCTCCGTC<br>R: AATAAGCTTTCAGTCTTCCCACTCATCATCATCC
pET15b-hN-WASP	subcloned	*pET15B*	*Xma*I/*Hind*III	
pFastBacHTA-hN-WASP	cloned	*pFastBacHTA*	*Eco*RI/*Hind*III	F: ATATGAATTCATGAGCTCCGTCCAG<br>R: ATTAAGCTTTCAGTCTTCCCACTC
pMALTEV-hN-WASP Frag-1 (1–100 aa)	cloned	*pMALTEV*	*Xma*I/*Hind*III	F: ATTCCCGGGTTATGAGCTCCGTC<br>R: ATTAAGCTTTTAATATACAAAGTTATTGTATAGCT
pMALTEV-hN-WASP Frag-2 (101–250 aa)	cloned	*pMALTEV*	*Xma*I/*Hind*III	F: ATTCCCGGGTTAATAGTCCTAGAGGAT<br>R: ATTAAGCTTTTATTCTCTGTCTTTAAGTTGTGCCT
pMALTEV-hN-WASP Frag-3 (251–319 aa)	cloned	*pMALTEV*	*Xma*I/*Xho*I	F: ATTCCCGGGTTACATCAAAAGTTA<br>R: ATATCTCGAGTTATGTGGGAGCTCTTGA
pMALTEV-hN-WASP Frag-4 (320–400 aa)	cloned	*pMALTEV*	*Xma*I/*Xho*I	F: ATTCCCGGGTTGCTGCACCTCCA<br>R: ATTAAGCTTTTATGGAACCTGATGGTCC
pMALTEV-hN-WASP Frag-5 (401–505 aa)	cloned	*pMALTEV*	*Xma*I/*Hind*III	F: ATTCCCGGGTTACTACTGCAG<br>R: AATAAGCTTTCAGTCTTCCCACTCATCATCATCC

Supplementary Table S1 (continued)

Name	From	Vector	restriction Enzyme site	PCR-primer (5' to 3')
pGEXTEV-hN-WASP Frag-5 (401–505 aa)	cloned	*pGEXTEV*	*XmaI/XhoI*	F: ATTCCCGGGTTACTACTGCAG<br>R: ATATCTCGAGTCAGTCTTCCCACTCATC
pFlag-CMV2-hPQBP1	Zhang et al. (2017) (8)	*pFlag-CMV2*	*Bam*HI/*Not*I	
pFlag-CMV2-hPQBP1$^{\Delta WW}$	subcloned	*pFlag-CMV2*	*Bam*HI/*Not*I	
pFlag-CMV2-hPQBP1^{W52A}	cloned	*pFlag-CMV2*	*Bam*HI/*Not*I	F: TTGGAGGGCCTACCACCAAGCGCGTACAAGGTGTTCGACCCTTCC<br>R: GGAAGGGTCGAACACCTTGTACGCGCTTGGTGGTAGGCCCTCCAA
pFlag-CMV2-hPQBP1^{W75A}	cloned	*pFlag-CMV2*	*Bam*HI/*Not*I	F: GCAGACACAGACCTTGTATCCGCGCTCTCCCCACATGACCCCAAC<br>R: GTTGGGGTCATGTGGGGAGAGCGCGGATACAAGGTCTGTGTCTGC
pFlag-CMV2-hPQBP1$^{W52, 75A}$	cloned	*pFlag-CMV2*	*Bam*HI/*Not*I	F: TTGGAGGGCCTACCACCAAGCGCGTACAAGGTGTTCGACCCTTCC<br>R: GGAAGGGTCGAACACCTTGTACGCGCTTGGTGGTAGGCCCTCCAA
pFlag-CMV2-hPQBP1^{Y65C}	subcloned	*pFlag-CMV2*	*Bam*HI/*Not*I	
pGEXTEV-hPQBP1	Zhang et al. (2017) (8)	*pGEXTEV*	*Bam*HI/*Not*I	
pGEXTEV-hPQBP1-N	Zhang et al. (2017) (8)	*pGEXTEV*	*Bam*HI/*Not*I	
pGEXTEV-hPQBP1-M	Zhang et al. (2017) (8)	*pGEXTEV*	*Bam*HI/*Not*I	

Supplementary Table S1 (continued)

Name	From	Vector	restriction Enzyme site	PCR-primer (5' to 3')
pGEXTEV-hPQBP1-C	Zhang et al. (2017) (8)	*pGEXTEV*	*Bam*HI/*Not*I	
pGEXTEV-hPQBP1$^{\Delta WW}$	subcloned	*pGEXTEV*	*Bam*HI/*Not*I	
pGEXTEV-hPQBP1^{W52A}	subcloned	*pGEXTEV*	*Bam*HI/*Not*I	
pGEXTEV-hPQBP1^{W75A}	subcloned	*pGEXTEV*	*Bam*HI/*Not*I	
pGEXTEV-hPQBP1^{W52,75A}	subcloned	*pGEXTEV*	*Bam*HI/*Not*I	
pGEXTEV-hPQBP1^{Y65C}	Liu et al. (2020) (42)	*pGEXTEV*	*Bam*HI/*Not*I	
